# Geometry-informed temperature band screening for crop microclimate management in a commercial tomato greenhouse

**DOI:** 10.3389/fpls.2026.1969599

**Published:** 2026-09-29

**Authors:** Xiaojing Liu, Jianlin Li, Hongsheng Lin, Shuhui Shi

**Affiliations:** 1 College of Sciences, Shenyang Institute of Engineering, Shenyang, China; 2 Osaka Central Advanced Mathematical Institute, Osaka Metropolitan University, Osaka, Japan; 3 Horticulture Program, Dalian Art College, Dalian, China

**Keywords:** crop environment, greenhouse tomato, microclimate modeling, Rayleigh–Ritz solution, resource efficiency, temperature band screening

## Abstract

Commercial greenhouses require temperature strategies that coordinate natural ventilation and screens with supplementary heating while maintaining a suitable crop environment. We evaluated a low input framework that combines a spectral geometry descriptor, one hour autoregressive prediction with exogenous inputs (ARX), temperature-conditioning degree-hour indices, vapor pressure deficit (VPD) exposure, and recorded vent, screen, and heating water data to screen temperature bands for field testing. The case study used 24,132 complete five minute interval starts from a 104 m × 68 m × 6.5 m Venlo tomato compartment operating at 1.25 plants/m^2^, 3.75 productive stems/m^2^, and an annual yield of approximately 45 kg/m^2^. The Rayleigh–Ritz solution gave *C_G_*= 7.188 m^2^, and the maximum single mode error was 4.26% across the numerical test grid. A preliminary fourth root scaling hypothesis mapped the declared reference half widths of 0.8–1.2 °C to candidate half widths of 0.97–1.45 °C; the 1.21 °C midpoint is an illustrative scenario conditional on the declared benchmark and reference half width. Alternative benchmarks produced multipliers of 1.047–1.290, confirming benchmark dependence. Within-site temporal holdout evaluation of ARX yielded RMSE 0.798 °C and *R*^2^ = 0.911; this result evaluates temporal prediction separately from geometry scaling. On the same observed temperature series, crop reference band membership increased from 21.02% at ±1.0 °C to 25.22% at ±1.21 °C, while the total degree-hour index decreased by 9.17%. The 9.17% value is a retrospective index difference rather than a measured energy saving. Only 28.38% of daytime records were within the 0.5–1.2 kPa VPD reference interval, showing that the prevailing moisture environment remained a constraint beyond temperature-band flexibility. The framework therefore supplies candidate bands and crop-environment criteria for controlled evaluation of energy use, plant responses, yield, and fruit quality.

## 1 Introduction

Greenhouse production protects crops from variable weather but may require substantial heating and cooling (Van Beveren et al., 2015; Katzin et al., 2020; Trépanier et al., 2025; Zhang et al., 2020; Nagarsheth et al., 2025). Natural ventilation and deployable screens can reduce this demand by modifying heat and moisture exchange before supplementary conditioning is used. Their practical value depends on crop temperature and humidity requirements. Temperature affects photosynthesis, respiration, assimilate allocation, flowering, fruit development, and transpiration, while relative humidity determines VPD and atmospheric demand (Castilla, 2013; Heuvelink, 2018; Marcelis et al., 1998; Shamshiri et al., 2018). Greenhouse geometry and vertical stratification further influence microclimate distribution and the performance of passive controls (Boulard et al., 1997; Castilla, 2013; Li and Willits, 2008; Teitel et al., 2003).

Detailed greenhouse studies use computational fluid dynamics, model predictive control (MPC), machine learning, and reinforcement learning to examine heat transfer, spatial conditions, actuator behavior, and resource objectives (Lin et al., 2021; Chen et al., 2022; Hu and You, 2022; Ajagekar et al., 2023; Mahmood et al., 2023; Wang et al., 2024; Narayanan et al., 2025; Yang et al., 2024; Odhiambo et al., 2020; Nagarsheth et al., 2025; Labrozzi et al., 2026). Recent work has coupled long short-term memory prediction with predictive control and has used analytic hierarchy process weighting within MPC to balance climate, water, and energy objectives (Benzzine et al., 2026, 2025). These methods support prediction or closed-loop decision making when suitable training data, actuator models, and objective weights are available. The present framework addresses the upstream task of defining a small set of candidate bands from routine records and is intended to complement later LSTM or MPC evaluation.

Routine climate-computer records are well suited to early screening because temperature, humidity, radiation, vent, and screen data are commonly available, whereas synchronized energy use, crop gas exchange, and treatment specific yield are less common (Li et al., 2021; de Oliveira et al., 2024). Real-time monitoring and automated detection of sensor abnormalities can improve input quality (Bicamumakuba et al., 2026a, b). Intervention data are then needed for causal evaluation; routine records can first narrow the bands taken into field testing.

This study combines four elements for that preliminary decision: a geometry descriptor, short term temperature prediction, crop environment exposure, and observed control operation. Weighted Hardy inequalities provide the mathematical basis for a compact spectral descriptor linking domain geometry with a weighted temperature error norm (Kufner and Persson, 2003). The descriptor is solved numerically and checked against a single mode approximation. An ARX model tests whether routine weather and temperature records support one hour prediction. Temperature and VPD describe crop exposure, while vent, screen, and heating water records provide operational context.

We examined whether greenhouse geometry and routinely recorded environmental data could be combined to generate and evaluate a limited family of temperature band scenarios for field testing. The objectives were to (i) formulate and numerically verify the geometry descriptor, (ii) evaluate the ARX model on a chronological holdout from the same compartment and crop cycle, (iii) generate a preliminary family of candidate bands from a declared benchmark and scaling hypothesis, and (iv) compare those candidates using degree-hour indices, temperature and VPD exposure, and operating records. Geometry calculation, temporal ARX evaluation, and operational associations were treated as distinct evidence levels; direct energy and crop measurements define the subsequent validation stage. The broader question was whether geometry-informed temperature flexibility could be screened without ignoring the accompanying crop moisture environment.

## 2 Materials and methods

### 2.1 Okawa greenhouse dataset and extracted variables

The dataset comprises 12 weekly climate computer exports collected by a coauthor during routine management of the Okawa Greenhouse, a commercial Venlo greenhouse with multiple compartments in Miyagi Prefecture, Japan. The records cover weeks 37–48 of 2021. The tomato growing compartment studied here measures 104 m long, 68 m wide, and 6.5 m high. Because tomato production runs from January to December, the September–December data represent the late stage of the annual crop cycle.

Large fruit tomatoes were grown on 1 m substrate slabs, with two plants and six productive stems per slab. At a row spacing of 1.6 m, planting density was approximately 1.25 plants/m^2^ and productive stem density was 3.75 stems/m^2^. Under routine management, annual yield was approximately 45 kg/m^2^, or 12 kg/stem/year. This value describes routine commercial production and was not measured under alternative temperature bands.

Greenhouse monitoring and environmental control used a Priva Connext 910 hardware and software platform supplied and serviced for commercial operation. The greenhouse was divided into independently managed climate compartments, each using dedicated environmental sensors. In the tomato growing compartment, the exported air temperature and relative humidity were the means of two dedicated temperature and relative-humidity measuring boxes. Both measuring boxes were maintained at the top of the crop canopy and adjusted vertically as the plants grew, so the averaged series represented the canopy-top air environment. A Priva outdoor weather sensor installed externally above the greenhouse roof recorded wind direction, wind speed, outdoor air temperature, rainfall, and solar radiation; only outdoor air temperature and solar radiation were used as ARX inputs. The climate-computer exports contained five minute observations, and variables were matched by label and channel descriptor because column order varied among weeks.

Five variables were available throughout the 12 weeks: outdoor temperature, solar radiation, indoor temperature, relative humidity, and CO_2_ concentration in the tomato growing compartment. W38–W44 also included heating supply water temperature, north and south vent opening, two screen deployment channels, and the actual heating setpoint. Vent values ranged from 0% (closed) to 100% (open); screen values ranged from 0% (retracted) to 100% (fully deployed). The ARX model used outdoor temperature, radiation, and the previous indoor temperature. Relative humidity and CO_2_ were used only to characterize the dataset. Vapor pressure deficit (VPD) was calculated using Equation 1:

(1) $$\text{VPD}=0.6108\exp\left(\frac{17.27T_{g}}{T_{g}+237.3}\right)\left(1-\frac{RH}{100}\right),$$

where *T_g_* is greenhouse air temperature in °C and RH is relative humidity in percent (Shamshiri et al., 2018). VPD was used as an indicator of atmospheric demand, not as a measurement of transpiration or stomatal conductance.

After duplicate removal, omission of one five hour gap without interpolation, and alignment to valid interval starts, 24,132 complete five minute intervals remained (99.75% temporal completeness). Detailed preprocessing checks are provided with the reproducibility materials. Site, crop-cycle, and measurement metadata are summarized in **Table 1**.

### 2.2 Temperature error domain and dimensionless weight

The tomato growing compartment was represented as

$\Omega =\left(0,L_{x}\right)\times \left(0,L_{y}\right)\times \left(0,H\right),$with *L_x_* = 104 m, *L_y_* = 68 m, and *H* = 6.5 m. Let *e*(***x***) denote a spatial temperature-error mode and define the dimensionless height *ξ* = *z*/*H*. Normalizing by structural height gives the same relative-height scale to geometries with different absolute heights; it does not imply that a vertical temperature profile was measured. The vertical weight was selected from the class of power-law weights used in weighted Hardy-type inequalities and was defined in Equation 2:

(2) $$w\left(\xi \right)=\xi^{-2\alpha },\;\;0<\alpha <\frac{1}{2}.$$

In the present formulation, *α* controls the relative emphasis placed on lower heights, and the restriction 0< *α* < 1/2 ensures that *w* is integrable on (0, 1). Because no vertical temperature profiles were available, neither the power-law form nor α was calibrated from measurements. The parameter *α* was therefore treated as a sensitivity parameter rather than a measured physical coefficient. Unlike a weight written directly as a power of dimensional *z*, *w* is dimensionless. The geometry descriptor is

(3) $$C_{G}={\left[\underset{e\in H_{0}^{1}\left(\text{Ω}\right),\;e\neq 0}{\inf}\frac{\int_{\Omega }{\left|\nabla e\right|}^{2}\;dx}{\int_{\Omega }w\left(z/H\right)e^{2}\;dx}\right]}^{-1}.$$

Both integrals have consistent dimensions, so *C_G_* has units of m^2^. Equation 3 is equivalent to the weighted Hardy-type inequality $\int_{\text{Ω}}we^{2}\;dx\leq C_{G}\int_{\text{Ω}}{\left|\nabla e\right|}^{2}\;dx$, and *C_G_* is its optimal constant for the stated domain, weight, and function space. The Rayleigh quotient and generalized principal eigenvalue provide the spectral characterization used here. Within this idealized comparison problem, a larger *C_G_* permits a larger weighted error amplitude for the same gradient energy. For a diffusion operator, an inverse first eigenvalue is also proportional to a slow spatial relaxation scale after division by diffusivity. This supplies a limited physical motivation for using *C_G_* as a geometry-dependent susceptibility descriptor, but it is not a heat-transfer model of glazing, ventilation, radiation, crop transpiration, or heating.

**Table 1:** Site, crop cycle, and measurement metadata.

Metadata item	Information
Facility	Okawa Greenhouse, commercial multiple compartment Venlo type greenhouse; year round operation
Location	Miyagi Prefecture, Japan
Tomato growing compartment geometry	104 m × 68 m × 6.5 m (length × width × structural height)
Crop cycle	Tomato transplanted in January; production ends in December
Cultivation system	Large fruit tomatoes grown on substrate slabs; two plants and six productive stems per slab of 1 m length
Crop density	Row spacing: 1.6 m; planting density: approximately 1.25 plants/m^2^; productive stem density: 3.75 stems/m^2^
Commercial annual yield	Approximately 45 kg/m^2^, equivalent to about 12 kg/stem/year; routine production context, not a treatment response
Monitoring phase	Weeks 37–48; late phase of the annual crop cycle
Monitoring and control platform	Priva Connext 910 greenhouse environmental-control hardware and software system
Primary temperature and RH series	Mean of two dedicated temperature and relative-humidity measuring boxes in the tomato growing compartment
Indoor measurement position	Both measuring boxes maintained at the top of the crop canopy and adjusted vertically as the plants grew
Outdoor weather sensor	Priva unit installed externally above the greenhouse roof; wind direction, wind speed, outdoor air temperature, rainfall, and solar radiation recorded
Detailed device and service metadata	Accuracy and resolution specifications, calibration documentation, individual device identifiers, and software-version information are retained in vendor-managed and site-specific technical records and were not available for public disclosure at the time of revision
Operational channels, W38–W44	Heating supply water temperature; north and south vent opening; deployment of screens 1 and 2; actual heating setpoint
Actuator coding	Vents: 0% fully closed, 100% fully open; screens: 0% fully open/retracted, 100% fully covering

The homogeneous Dirichlet space was chosen to produce a well-posed, reproducible first-eigenvalue comparison with a finite characteristic scale. It does not assert that temperature error is zero on every physical greenhouse surface. Other boundary models would produce different descriptors and require thermal boundary data that were unavailable here.

The denominator in Equation 3 normalizes the trial function by its weighted *L*^2^ norm. Consequently, *C_G_* is an inverse eigenvalue and is not numerically comparable with an unnormalized integral indicator. Only the dimensionless ratio *C_G_*_,site_/*C_G_*_,b_ enters the scaling rule below.

In the absence of simultaneous vertical temperature profiles, *α* was treated as a sensitivity parameter rather than an estimated coefficient. The interval 0.20–0.35 represents moderate power-law weighting scenarios within the admissible range 0< *α* < 0.5; the midpoint *α* = 0.30 was used solely as a reporting baseline. Linear or exponential weights were not compared because the available single-position record could not calibrate or discriminate among vertical weighting families. Multi-height measurements are required to select both the functional form and parameter empirically.

### 2.3 Rayleigh–Ritz solution and single mode check

Because *w* depends only on *z*/*H*, the rectangular comparison problem separates in the horizontal and vertical coordinates. The first horizontal sine mode minimizes the horizontal gradient contribution; any higher horizontal index adds a positive Laplacian eigenvalue and cannot produce the minimum. The vertical component was therefore expanded as shown in Equation 4:

(4) $$e\left(x,y,z\right)=\sin\left(\frac{\pi x}{L_{x}}\right)\sin\left(\frac{\pi y}{L_{y}}\right)\sum_{n=1}^{N}c_{n}\sin\left(\frac{n\pi z}{H}\right).$$

Substitution into (3) gives a symmetric generalized eigenvalue problem. Thus, the horizontal mode is exact within the stated rectangular Dirichlet formulation, whereas the vertical expansion is truncated numerically. We used *N* = 24 vertical modes and 800 point Gauss–Legendre quadrature. Convergence was checked with *N* = 4, 8, 12, 24, and 48. Detailed matrix construction is provided in **Supplementary Methods S1**–**S3**, and the convergence values are reported in **Supplementary Table S2**.

For comparison, retaining only the first vertical mode gives Equation 5:

(5) $$C_{G,1m}=\frac{2I_{\alpha }}{{\left(\frac{\pi }{L_{x}}\right)}^{2}+{\left(\frac{\pi }{L_{y}}\right)}^{2}+{\left(\frac{\pi }{H}\right)}^{2}},\;\;I_{\alpha }=\int_{0}^{1}\xi^{-2\alpha }\sin^{2}(\pi \xi )\;d\xi .$$

This expression is dimensionally consistent but remains an approximation. Its error was evaluated for five rectangular geometries and *α* = 0.20, 0.30, and 0.35 rather than assumed negligible.

### 2.4 Geometry scaling as a preliminary screening hypothesis

The spectral descriptor does not itself prescribe a temperature band. To convert it into a testable scenario, we introduced the phenomenological objective in Equation 6:

(6) $$J\left(\delta \right)=b\frac{C_{G}}{\delta^{2}}+g\delta^{2},\;\;b>0,g>0.$$

The coefficients *b* and *g* make the terms commensurate. The factor *C_G_*/*δ*^2^ represents a geometry-weighted susceptibility normalized by the square of the allowed temperature deviation: it becomes smaller when a wider band is tolerated and larger for a geometry with a larger spectral susceptibility. The term *δ*^2^ is a symmetric quadratic penalty on allowing greater temperature deviation. These are screening preferences, not measured heat demand and crop damage functions. Differentiation gives $dJ/d\delta =-2bC_{G}\delta^{-3}+2g\delta$ and the stationary condition gives $\delta^{4}=bC_{G}/g$; the complete derivation is provided in **Supplementary Methods S4**.

The fourth-root exponent follows from balancing *bC_G_*/*δ*^2^ against *gδ*^2^; it is not derived directly from greenhouse heat transfer or crop physiology. More generally, $bC_{G}\delta^{-p}+g\delta^{q}$ would give a geometry exponent of 1/(*p* + *q*). The present choice therefore defines a preliminary, transparent scaling hypothesis to be tested, not a validated greenhouse design or control rule. If *b*/*g* is held constant between two geometries, the stationary half widths satisfy

(7) $$\frac{\delta_{site}}{\delta_{b}}={\left(\frac{C_{G,site}}{C_{G,b}}\right)}^{1/4}.$$

Equation 7 defines the fourth root geometry multiplier under that hypothesis. The Okawa calculation was normalized against a declared benchmark measuring $\sqrt{1000}\;\text{m}\times \sqrt{1000}\;\text{m}\times 4.5\;\text{m}$. The benchmark is a reproducible reference, not a standard greenhouse or an estimated crop optimum. Its dimensions and reference half width must therefore be reported as a pair.

Benchmark dependence was tested using square plan areas of 500, 1000, and 2000 m^2^ and heights of 4.0, 4.5, and 6.0 m. The reference half width was fixed at 1.0 °C to isolate the effect of benchmark geometry.

Because *b*/*g* could not be estimated from the records, a declared half width of 1.0 °C was used as the central comparison, and a narrow ±20% sensitivity family (0.8–1.2 °C) was propagated through Equation 7. This family was not inferred from the Priva controller, an industry standard, or crop physiology. Replacing the reference family would linearly rescale the candidate family. The 1.0 °C midpoint generated the illustrative site scenario. The provenance and interpretation of the screening inputs are summarized in **Table 2**.

**Table 3:** Summary of the 84 day Okawa dataset used for interval based analysis. Values in parentheses are ranges.

Period	Intervals	Outdoor temperature, °C	Greenhouse temperature,	Solar radiation, MJ/m^2^/day	RH, %	VPD, kPa
W37–W40 early autumn	8064	19.38 (13.0–27.4)	20.76 (15.3–29.0)	12.42	89.33 (65.0–96.0)	0.288
W41–W44 mid autumn	8004	12.71 (4.0–25.9)	18.84 (14.1–29.9)	10.12	89.58 (63.0–96.0)	0.254
W45–W48 late autumn/early winter	8064	8.38 (−0.4–19.5)	18.36 (14.0–25.9)	8.08	85.96 (68.0–95.0)	0.305
All W37–W48	24132	13.49 (−0.4–27.4)	19.32 (14.0–29.9)	10.21	88.29 (63.0–96.0)	0.282

### 2.5 Data processing and ARX temperature model

Records were sorted by timestamp, duplicates were removed, and missing intervals were left unfilled. Solar radiation was integrated over five minute intervals for daily summaries; indoor temperature, outdoor temperature, and radiation were averaged hourly. One hour prediction pairs were retained only for contiguous timestamps.

An autoregressive model with exogenous inputs (ARX) tested whether the routinely available variables supported a transparent one hour temperature forecast. Although recurrent neural networks and LSTM- based predictive control can represent more complex nonlinear dynamics (Oh et al., 2024; Yuan et al., 2024; Benzzine et al., 2026), the ARX structure in Equation 8 was chosen for its low input requirements and interpretable coefficients; it was not intended as an alternative controller:

(8) $$T_{g,k+1}=\phi_{1}T_{g,k}+\phi_{2}T_{o,k}+\phi_{3}R_{k}+\phi_{4}\sin\left(\frac{2\pi q_{k}}{24}\right)+\phi_{5}\cos\left(\frac{2\pi q_{k}}{24}\right)+\phi_{0}+\epsilon_{k},$$

where *T_g_*,*_k_* is indoor air temperature, *T_o_*,*_k_* is outdoor air temperature, *R_k_* is mean solar radiation, and *q_k_* is hour of day. Weeks 37–44 were used for calibration and the chronologically later W45–W48 records were held out for evaluation. This within-site temporal holdout represents the same greenhouse compartment and crop cycle; external validation across sites and seasons remains a separate step. The chronological split avoided leakage from random allocation and tested transfer to thecolder end of the monitored period. Performance was evaluated using RMSE, MAE, bias, and *R*^2^.

We compared the ARX model with persistence, ${\hat{T}}_{g,k+1}=T_{g,k}$, and an AR(1) model, ${\hat{T}}_{g,k+1}=aT_{g,k}+b$. Metrics were also calculated separately for W45–W48. Fitted coefficients were retained, and ordered residuals were summarized by their distribution, Durbin–Watson statistic, and autocorrelation at lags 1–24 h. Because the geometry descriptor *C_G_* is not an ARX input, these metrics characterize the temporal prediction component; prospective cross-geometry comparisons address the geometry-to-band scaling.

### 2.6 Temperature conditioning degree-hour indices and band settings

Measured indoor temperature was used to calculate temperature-conditioning degree-hour indices. Daytime (06:00–18:00) used *T*_ref_ = 25 °C and night (18:00–06:00) used *T*_ref_ = 18 °C, within the tomato ranges summarized by Shamshiri et al. (2018). Cultivar- and stage-specific optima were unavailable. The fixed pair therefore provided a consistent literature-guided exposure reference for this late-stage crop period, not a measured physiological optimum or the climate-computer setpoints. It can be replaced by time-varying references for cultivar, crop stage, radiation, or fruit load. The heating degree-hour index (HDH) was calculated using Equation 9.

(9) $$HDH=\sum_{k}\max\left[0,T_{\text{ref},k}-\delta_{k}-T_{g,k}\right]\Delta t,$$

The cooling degree-hour index (CDH) was calculated using Equation 10:

(10) $$CDH=\sum_{k}\max\;\left[0,T_{g,k}-T_{\text{ref},k}-\delta_{k}\right]\Delta t,$$

where Δ*t* = 5/60 h and *δ* is the half width of $\left[T_{\text{ref}}-\delta ,\;T_{\text{ref}}+\delta \right]$. Missing intervals were omitted, and all strategies used the same 24,132 interval starts. These indices quantify accumulated temperature excursions beyond the selected band (Bailey and Seginer, 1989); they are not measurements or estimates of energy use.

Four symmetric scenarios were compared: fixed narrow (± 0.5 °C), declared reference (± 1.0 °C), the illustrative geometry-scaled candidate (± 1.21 °C), and fixed wide (± 1.5 °C).

For the illustrative and reference comparison, degree-hour contributions were first aggregated by calendar day so that resampling preserved all dependence among the 288 five minute observations within a day. The 83 complete days were resampled with replacement 20,000 times using a fixed seed. Because calendar-day resampling does not preserve dependence between consecutive days, serial dependence was assessed from the lag-1 autocorrelation of the paired daily index difference and a seven-day moving-block bootstrap was added. Only blocks of consecutive complete days were eligible. The 2.5th and 97.5th percentiles of each bootstrap distribution were reported. Both intervals describe sampling variation in a deterministic index that decreases monotonically as the band widens.

### 2.7 Crop environment and control variables

Each five minute record was classified relative to the ±1.0 °C and ±1.21 °C crop reference bands. VPD was classified as below, within, or above the 0.5–1.2 kPa interval summarized by Shamshiri et al. (2018). As with the temperature references, this was a literature-guided screening interval applied consistently to the late-stage record, not a measured optimum for the cultivar or crop stage. Joint exposure required membership in both the temperature band and the VPD interval. Results were separated into day (06:00– 18:00) and night (18:00–06:00) because the pooled distribution obscures daytime atmospheric demand. These measures describe the canopy-top air environment; physiological responses were not measured.

Natural vents and deployable screens were treated as passive control channels, and heating water temperature represented supplementary conditioning. Their relationships with weather and greenhouse temperature were used to check operational consistency. The actuator states arose from the existing controller and were not treated as responses to an alternative temperature band.

### 2.8 Secondary operational context checks

The W38–W44 operating channels provided physical context for the degree-hour indices. After the same duplicate and missing data rules were applied, 14,052 of 14,112 expected five minute records were complete (99.57%). Hourly aggregation and removal of partial hours and the incomplete day left 48 complete days (1152 hourly records); the daytime cooling analysis used 576 records.

Heating- and cooling-band excursions were the nonintegrated terms in the HDH and CDH equations. Mean vent opening was the average of the north and south channels. Spearman correlations compared the heating-band excursion with supply water temperature and the daytime cooling-band excursion with mean vent opening. Supply water temperature was also compared with the positive difference between the heating setpoint and outdoor temperature. Descriptive intervals were obtained from 20,000 calendar-day resamples, which preserved within-day observations but did not fully model dependence across consecutive days. Night screen deployment was summarized above and below an outdoor threshold of 10 °C. These tests assessed operational consistency rather than energy use or causal actuator effects.

A heating sensitivity analysis replaced the crop reference temperature with the recorded heating setpoint *T*_heat,_*_k_*, as shown in Equation 11:

(11) $$HDH_{\text{set},s}=\sum_{k}\max\;\left[0,T_{\text{heat},k}-\delta_{s,k}-T_{g,k}\right]\Delta t,$$

where *s* denotes the declared reference or illustrative geometry-scaled scenario. The analysis used 13,824 five minute records from the same 48 complete days and 20,000 calendar day resamples. Cooling was not analyzed because no recorded cooling setpoint was available.

An ARX sensitivity analysis tested whether actuator records improved one hour prediction during W38–W44. Models were calibrated on W38–W42 and evaluated on W43–W44. The base specification used previous indoor temperature, outdoor temperature, radiation, and diurnal terms. The second model added mean vent opening and two screen channels; the third also added heating water temperature.

Predictor redundancy was assessed in the W38–W42 calibration set using variance inflation factors (VIF), the condition number of the standardized predictor matrix, and the maximum absolute Pearson correlation. These diagnostics cannot identify causal actuator effects because the actuator states depend on the existing controller.

### 2.9 Parameter sensitivity analysis

With the compartment dimensions fixed, *α* was varied from 0.20 to 0.35 to evaluate its effect on *C_G_*. Reference half widths from 0.8 to 1.2 °C were propagated through the fourth root geometry multiplier. The additional nine case benchmark grid separated sensitivity to declared benchmark area and height from sensitivity to *α*. A symmetric half width sweep from 0.4 to 2.0 °C then quantified the monotonic response of HDH, CDH, and the total degree-hour index.

### 2.10 Software and reproducibility

Calculations were performed in Python 3.12.13 using NumPy 2.5.2, pandas 2.2.3, openpyxl 3.1.5, and Matplotlib 3.11.1. The submitted script records numerical settings, resampling seeds, convergence checks, exposure summaries, and aggregate results. A machine readable requirements file lists all third party dependencies.

### 2.11 Screening and application workflow

The workflow has four stages (**Figure 1**). Stage 1 uses greenhouse dimensions and a declared reference family to calculate *C_G_* and generate candidate bands under the preliminary scaling hypothesis. Stage 2 evaluates the local ARX model by a within-site temporal holdout and summarizes degree-hour indices, temperature, RH, and VPD. Stage 3 checks whether vent, screen, and heating records agree with their expected operating roles. Stage 4 tests the retained bands using controlled measurements of energy, water, crop physiology, yield, and quality. The first three stages were evaluated here; field validation remains future work. The complete application sequence is listed in **Supplementary Methods S12**.

**Figure 1:**
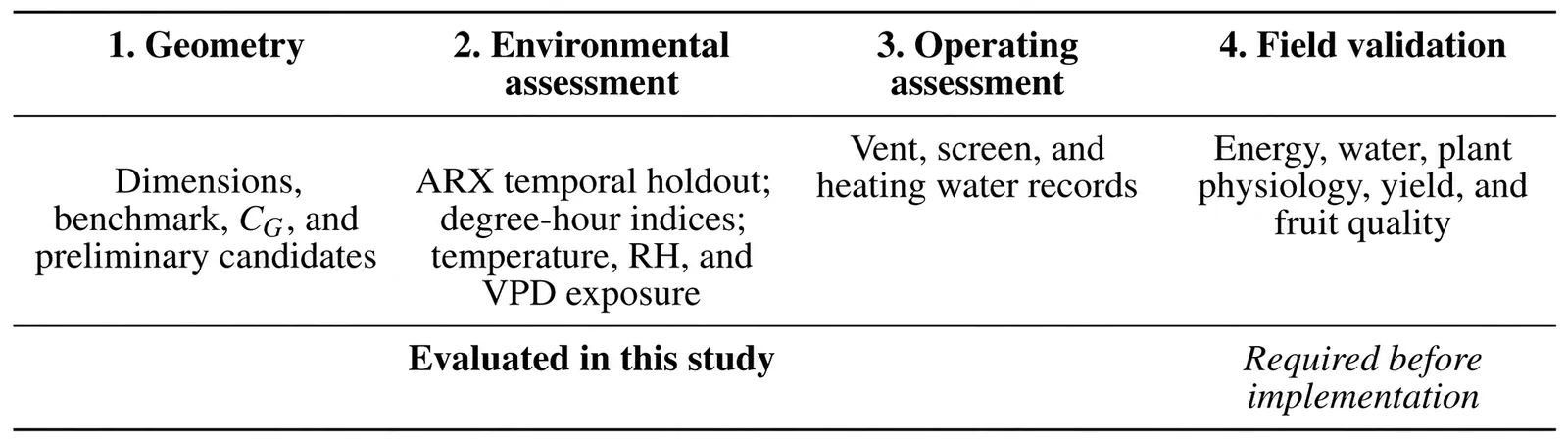
Temperature band screening stages. The present study covers geometry, environmental assessment, and operating assessment; controlled field validation is required before implementation.

### 2.12 Evidence levels and interpretation

Four evidential levels were defined. First, mode refinement, quadrature, and the single-mode comparison verified the numerical calculation of *C_G_* within the stated mathematical formulation. Second, the chronological ARX holdout evaluated one hour temporal prediction in the same compartment and crop cycle. Third, actuator associations assessed whether the recorded operating channels were qualitatively consistent with expected roles. These three levels support candidate generation and local assessment but remain distinct from validation of the fourth-root relationship. The fourth level is prospective comparison of candidate bands across greenhouse geometries using spatial climate measurements, metered energy, and crop responses.

## 3 Results

### 3.1 Seasonal environmental conditions

From W37–W40 to W45–W48, mean outdoor temperature decreased from 19.38 to 8.38 °C and mean greenhouse temperature decreased from 20.76 to 18.36 °C (**Table 3**; **Figure 2**). Mean daily solar radiation declined from 12.42 to 8.08 MJ/m^2^/day. Across all 12 weeks, mean relative humidity was 88.29% and mean VPD was 0.282 kPa (5th–95th percentile: 0.095–0.691 kPa), indicating a humid canopy environment. Under the declared reference ±1.0°C band, both HDH and CDH were present early in the record, but HDH became dominant as conditions cooled (**Figure 3**). The total degree-hour index increased from 772.36 °C h in W37–W40 to 1483.97 °Ch in W45–W48, while CDH was nearly zero in the final period.

**Table 2:** Provenance and interpretation of the screening inputs.

Input	Baseline or range	Interpretation
Crop reference temperature	25 °C day; 18 °C night	Literature guided values used only for retrospective sensitivity (Shamshiri et al., 2018); not the recorded control law.
Vertical weight *α*	0.30; sensitivity 0.20–0.35	Dimensionless sensitivity parameter; 0.30 is the midpoint reporting baseline, not a fitted value.
Rayleigh–Ritz discretization	24 modes; 800 quadrature points	Numerical solution of (3); checked against mode refinement.
Normalization benchmark	1000m^2^ square plan; 4.5 m height	Declared comparison geometry; not a design recommendation.
Reference half width	0.8–1.2 °C	Declared ±20% sensitivity around the 1.0 °C comparison; not an observed controller setting or crop optimum.
Illustrative site scenario	1.21 °C	Midpoint reference multiplied by the fourth root geometry multiplier; a preliminary candidate, not an optimized band.

**Figure 2:**
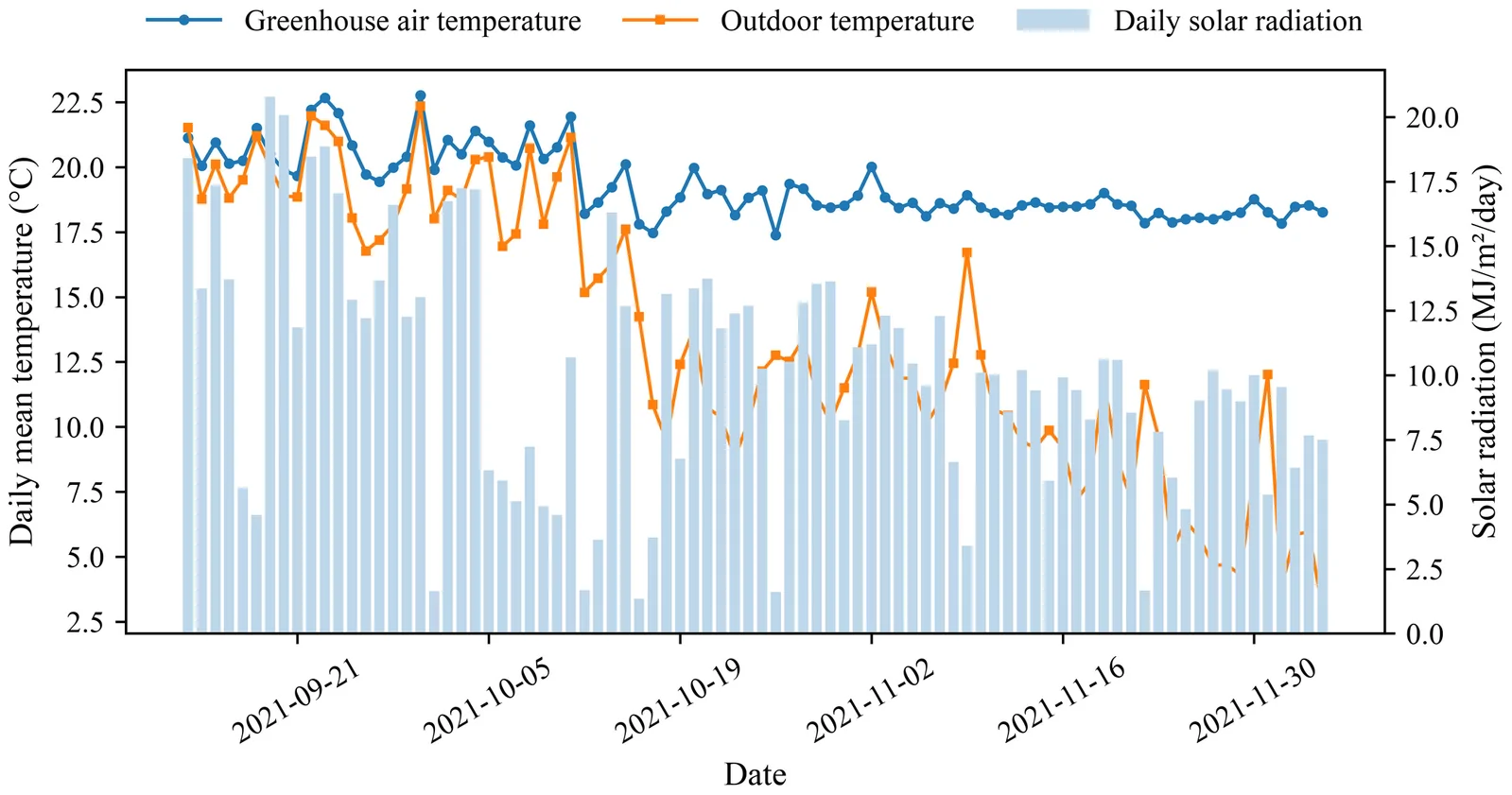
Daily mean outdoor temperature, air temperature in the tomato growing compartment, and solar radiation across the 12 week dataset.

**Figure 3:**
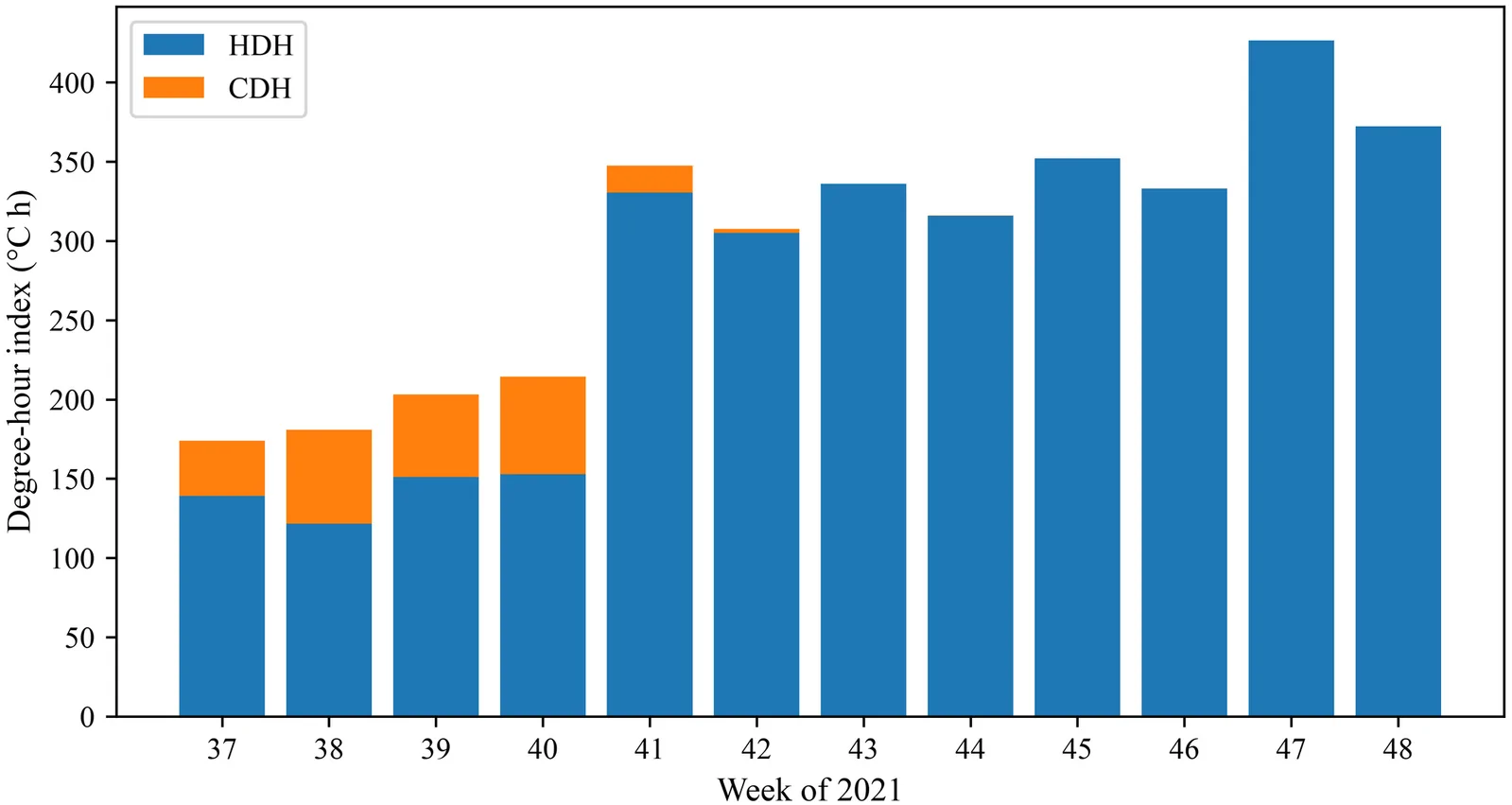
Weekly heating and cooling degree-hour indices under the declared reference ±1.0 ^°^C temperature band.

### 3.2 Crop relevant temperature and VPD exposure

Across 24,132 interval starts, 21.02% of observations were within the ±1.0 °C crop reference band and 25.22% were within the ±1.21 °C band (**Table 4**). Most observations were below the day and night reference bands, especially during W45–W48.

**Table 4:** Observed crop environment exposure by monitoring period.

Period	Temperature within ±1.0, %	Temperature within ±1.21, %	VPD 0.5–1.2 kPa, %	Joint ±1.0 and VPD, %	Joint ±1.21 and VPD, %
W37–W40	31.89	37.51	18.79	9.08	10.28
W41–W44	22.91	26.64	15.37	8.48	9.86
W45–W48	8.26	11.52	8.68	1.34	1.85
All W37–W48	21.02	25.22	14.28	6.29	7.32

Temperature membership uses literature guided day and night references of 25/18 °C. The VPD interval of 0.5–1.2 kPa is a literature guided screening range, not a measured physiological optimum for this crop.

Only 14.28% of all observations were within the 0.5–1.2 kPa VPD interval; 85.65% were below 0.5 kPa. The corresponding membership was 28.38% during the day and 0.15% at night (**Table 5**). Daytime joint temperature and VPD membership increased from 12.55% at ±1.0 °C to 14.61% at ±1.21 °C. The wider temperature band therefore did not remove the moisture constraint; the joint distributions are shown in **Supplementary Figure S1**.

**Table 5:** VPD and joint temperature and VPD exposure stratified by time of day.

Period	Records	VPD< 0.5 kPa, %	VPD 0.5–1.2 kPa, %	VPD *>* 1.2 kPa, %	Joint ±1.0, %	Joint ±1.21, %
Daytime	12077	71.47	28.38	0.15	12.55	14.61
Night	12055	99.85	0.15	0.00	0.02	0.02
All 24 h	24132	85.65	14.28	0.07	6.29	7.32

Daytime was 06:00–18:00 and night was 18:00–06:00. The categories describe atmospheric exposure and do not measure plant response.

### 3.3 ARX calibration and within-site temporal holdout

The hourly ARX model achieved an RMSE of 0.788 °C and *R*^2^ = 0.946 during calibration (W37–W44). During the W45–W48 temporal holdout, its RMSE was 0.798 °C, MAE was 0.620 °C, bias was 0.029 °C, and *R*^2^ was 0.911. Its holdout RMSE was 22.7% lower than persistence and 21.5% lower than AR(1) (**Table 6**), showing that the weather and diurnal terms added predictive information within the monitored site and crop cycle.

**Table 6:** Performance of the greenhouse temperature models with a prediction horizon of one hour.

Model	Period	Records	RMSE, °C	MAE, °C	Bias, °C	*R* ^2^
ARX	Calibration: W37–W44	1338	0.788	0.588	0.000	0.946
ARX	Temporal holdout: W45–W48	672	0.798	0.620	0.029	0.911
Persistence	Temporal holdout: W45–W48	672	1.033	0.747	−0.001	0.852
AR(1)	Temporal holdout: W45–W48	672	1.017	0.748	0.063	0.856

Holdout RMSEs for the individual weeks were 0.811, 0.785, 0.765, and 0.831 °C for W45–W48, respectively; *R*^2^ ranged from 0.874 to 0.931. The fitted coefficients are reported in **Supplementary Table S3**. Holdout residuals had a standard deviation of 0.798 °C and a Durbin–Watson statistic of 1.392. Residual autocorrelation was 0.302 at 1 h and 0.486 at 24 h (**Supplementary Table S4**; **Figure 4**), indicating remaining short-term and diurnal structure. The model therefore provides useful within-site one hour prediction, while the residual structure identifies a target for expanded dynamic modeling. The observed–predicted comparison and residual autocorrelation are shown in **Figure 4**. Together with **Table 6**, these diagnostics document the temporal prediction component and its remaining structure.

**Figure 4:**
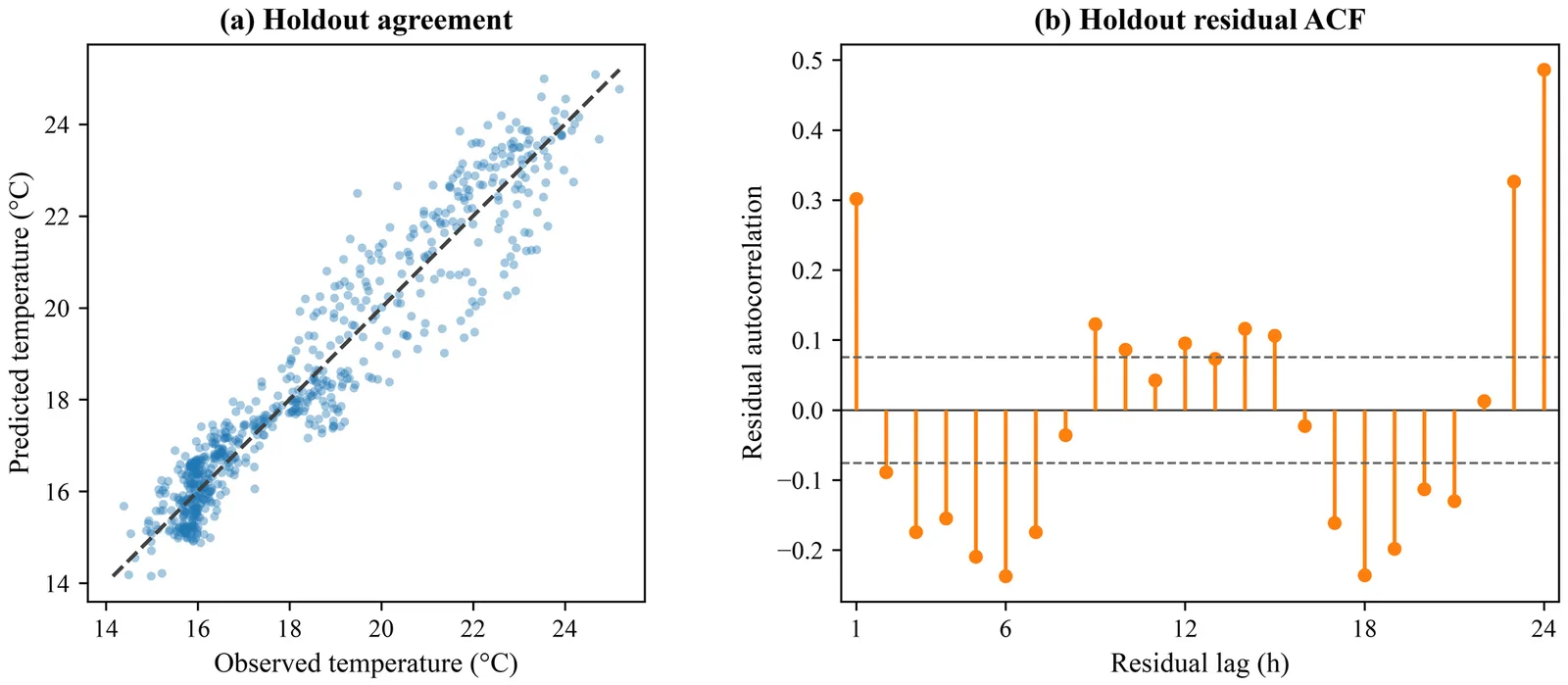
ARX diagnostics for the W45–W48 within-site temporal holdout. **(a)** Predicted versus observed hourly air temperature; the dashed line is 1:1. **(b)** Autocorrelation√ of ordered one-step residuals for lags 1–24 h; dashed horizontal lines show the approximate ±1.96/*n* bounds as visual reference only.

### 3.4 Geometry descriptor and numerical verification

For the measured dimensions and *α* = 0.30, the Rayleigh–Ritz solution with 24 vertical modes gave *C_G_* = 7.188 m^2^; the single vertical-mode value was 6.988 m^2^, an underestimate of 2.79% (**Table 7**). Increasing the vertical basis from 24 to 48 modes changed *C_G_* by less than 0.001%. These checks establish numerical stability within the stated boundary and weighting assumptions; experimental assessment of the corresponding spatial temperature field requires multi-location and multi-height observations.

**Table 7:** Sensitivity of the numerical geometry descriptor and fourth root geometry multiplier to the vertical weighting parameter.

*α*	Site *C_G_*, m^2^	Benchmark *C_G_*_,b_, m^2^	Site/benchmark ratio	Fourth root geometry multiplier	Single mode underestimate, %
0.20	5.922	2.764	2.142	1.210	1.13
0.25	6.508	3.039	2.142	1.210	1.85
0.30	7.188	3.357	2.141	1.210	2.79
0.35	7.983	3.729	2.140	1.210	3.99

**Table 7** reports the Okawa geometry and its paired benchmark. In the separate 15-case scan, the maximum single mode underestimate was 4.26% across five geometries and three *α* values. The 4.26% value is the approximation error for the numerical descriptor.

At *α* = 0.30, the benchmark geometry gave *C_G_*_,b_ = 3.357 m^2^. The ratio between the Okawa and benchmark values was 2.141, and the fourth root geometry multiplier under the preliminary scaling hypothesis was 1.210. Recomputing both geometries across *α* = 0.20–0.35 changed the unrounded multiplier only from 1.20982 to 1.20956 (**Table 7**); the vertical weighting affected the two descriptors similarly and largely cancelled in their ratio. The declared reference half widths of 0.8–1.2 °C mapped to an illustrative Okawa candidate family of 0.97–1.45 °C. The midpoint candidate was ±1.21 °C.

Alternative benchmark geometries produced multipliers of 1.047–1.290 (**Supplementary Table S1**). Plan area had a smaller effect than benchmark height over the tested grid. The baseline 1000 m^2^, 4.5 m benchmark gave 1.210, confirming that the 1.21 °C midpoint depends directly on the declared benchmark and reference half width.

### 3.5 Retrospective temperature band sensitivity

All four scenarios were evaluated on the same 24,132 five minute interval starts (**Table 8**). Heating accounted for 93.6% of the declared-reference total. Increasing *δ* from 1.0 to 1.21 °C reduced HDH from 3335.85 to 3047.09 °C h and CDH from 227.77 to 189.66 °C h, giving a 9.17% lower total index.

**Table 8:** Retrospective degree-hour index sensitivity to symmetric temperature band half width.

Scenario	*δ*, °C	HDH, °C h	CDH, °C h	Total, °C h	Heating share, %	Change from *δ* = 1.0, %
Fixed narrow	0.50	4078.16	338.76	4416.92	92.33	+23.94
Declared reference	1.00	3335.85	227.77	3563.62	93.61	0.00
Illustrative geometry scaled	1.21	3047.09	189.66	3236.75	94.14	−9.17
Fixed wide	1.50	2673.46	143.20	2816.66	94.92	−20.96

The lag-1 autocorrelation of the daily paired difference was 0.526. The calendar-day bootstrap interval was 8.66–9.68%, whereas the seven-day moving-block interval, which retained part of the between-day dependence, was wider at 8.41–10.14%. The median daily difference was 4.03 °C h. Because all calculations used the same observed temperature trajectory, the 9.17% value quantifies deterministic index sensitivity to a wider tolerance rather than a counterfactual temperature simulation or measured energy saving.

### 3.6 Operational context

The W38–W44 subset contained 14,052 complete five minute records. Heating supply water temperature ranged from 17 to 61 °C. The recorded heating setpoint ranged from 12 to 17 °C (mean 15.82 °C; median 16.0 °C), demonstrating that the fixed 25/18 °C crop reference pair differed from the climate computer’s operating setpoints.

The hourly heating-band excursion was positively associated with supply water temperature (**Figure 5a**; Spearman *ρ* = 0.344, daily cluster 95% interval 0.251–0.428). Supply water temperature was also associated with the positive difference between the heating setpoint and outdoor temperature (*ρ* = 0.415, interval 0.298–0.509). The daytime cooling-band excursion was positively associated with mean vent opening (**Figure 5b**; *ρ* = 0.451, interval 0.313–0.559).

**Figure 5:**
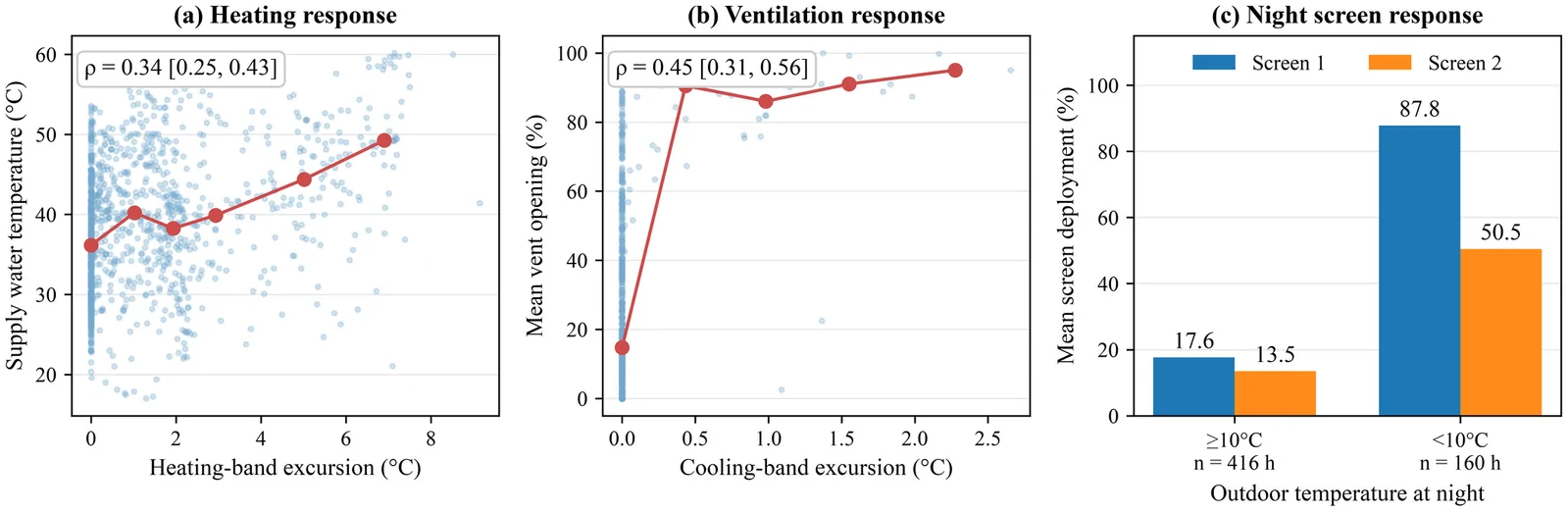
Operational context checks for 48 complete days in W38–W44. **(a)** Hourly heating-band excursion for the declared reference band and heating supply water temperature. **(b)** Daytime cooling-band excursion for the declared reference band and mean vent opening. Red points and lines show medians within each range; *ρ* values are Spearman correlations with descriptive calendar-day resampling intervals. **(c)** Mean night screen deployment above and below an outdoor temperature of 10 ^°^C. Screen coding ranges from 0% (retracted) to 100% (fully deployed).

At night, mean deployment of screens 1 and 2 was 17.6% and 13.5% when outdoor temperature was at least 10 °C, compared with 87.8% and 50.5% during 160 colder hours (**Figure 5c**). These relationships were consistent with expected operation. Conversion to energy units would require water flow, valve position, boiler input, electricity, and fuel measurements.

In the W43–W44 temporal holdout, the base ARX achieved RMSE 0.939 °C and *R*^2^ = 0.934 (**Table 9**). Adding vents and screens increased RMSE to 0.992 °C; adding heating water temperature gave RMSE 0.951 °C. The actuator channels did not improve prediction across this split.

**Table 9:** ARX sensitivity with actuator inputs during the period with operating channel data.

Model	Added operating channels	RMSE, °C	MAE, °C	*R* ^2^
Base ARX	None	0.939	0.677	0.934
Passive channel ARX	Mean vent opening; screen 1; screen 2	0.992	0.768	0.926
Passive and heating ARX	Passive channels; heating water temperature	0.951	0.728	0.932

All models were calibrated on W38–W42 and evaluated on W43–W44 (*n* = 311). The comparison assesses predictive contribution; causal actuator effects require intervention data.

The predictor condition number rose from 6.92 in the base model to 9.05 with passive channels and 9.12 after heating water temperature was added. The two screen channels were strongly correlated (|*r*| = 0.933; VIF 9.25–9.91), and previous indoor temperature had VIF above 10 in all models. These findings are consistent with predictor redundancy. In addition, all actuator states were generated by the existing controller in response to the same climate, producing endogeneity in the observational ARX fit. The lower holdout accuracy after adding channels therefore describes predictive redundancy under the existing control regime rather than the physical efficacy of the actuators. Full diagnostics are reported in **Supplementary Tables S5** and **Supplementary Data Sheet S6**.

The recorded setpoint analysis used 13,824 observations from 48 complete days. Indoor temperature was below the heating setpoint in 9.67% of records. The *δ* = 1.0 °C tolerance produced 21.06 °C h and a 2.64% violation rate; the *δ* = 1.21 °C tolerance produced 14.86 °C h and a 2.37% violation rate. The relative difference was 29.40% (daily cluster interval 23.40–37.80%). The heating-band excursion was weakly associated with supply water temperature (*ρ* = 0.129, interval 0.077–0.178). The large relative change reflects small absolute totals and infrequent violations.

### 3.7 Band width sensitivity

Increasing the symmetric half width from 0.4 to 2.0 °C reduced both HDH and CDH but allowed greater deviation from the crop reference. The lower indices therefore represent greater thermal tolerance and must be evaluated together with crop response.

The HDH curve declined more steeply than the CDH curve (**Figure 6**). The total degree-hour index fell rapidly as the half width increased from 0.4 to approximately 1.0 °C and more gradually thereafter. Identification of an operational optimum would additionally require crop-response and metered-energy measurements.

**Figure 6:**
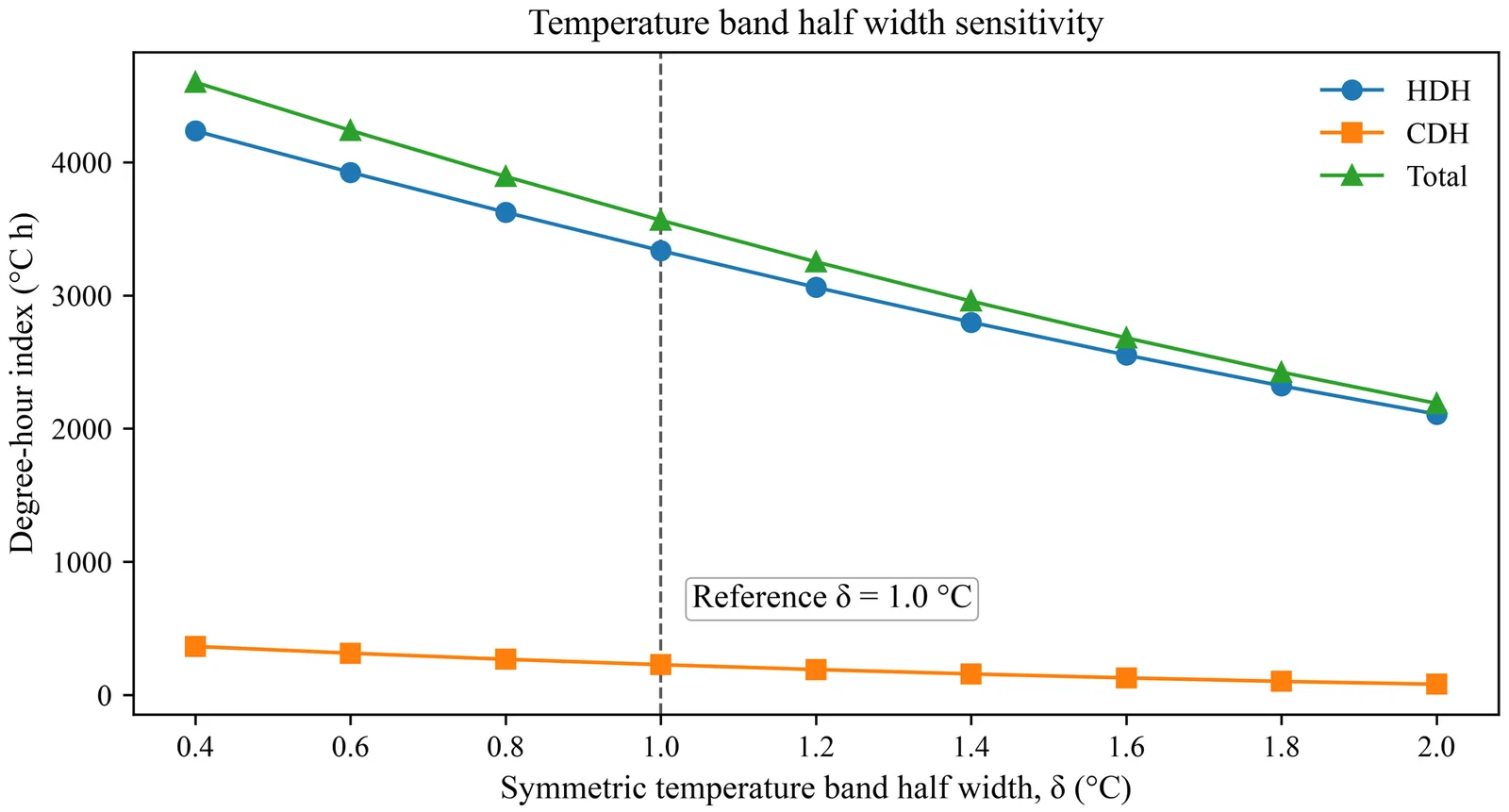
Sensitivity of heating, cooling, and total degree-hour indices to the symmetric temperature band half width. The vertical dashed line indicates the declared reference *δ* = 1.0 ^°^C half width.

## 4 Discussion

### 4.1 Main findings

The method reduced a broad control question to a set of preliminary temperature-band candidates for field testing. The spectral calculation was numerically stable within its assumptions, and the one hour ARX model retained useful accuracy during a four-week within-site temporal holdout, although residual autocorrelation remained. Using the declared benchmark and fourth-root hypothesis, the geometry multiplier mapped the 0.8–1.2 °C reference family to 0.97–1.45 °C. The conditional 1.21 °C midpoint provides a testable scenario under the stated benchmark, reference half width, and phenomenological objective rather than a universal optimum.

On the observed temperature series, increasing the half width from 1.0 to 1.21 °C lowered the total degree-hour index by 9.17%. This value quantifies retrospective index sensitivity; evaluation of fuel or electricity savings requires direct energy measurements under an applied control treatment. The exposure analysis also showed that temperature alone is insufficient: only 28.38% of daytime records were within the 0.5–1.2 kPa VPD interval. A candidate band must therefore be evaluated together with humidity control and crop response.

### 4.2 Use in greenhouse design and operation

During preliminary design, the method supports exploratory comparison of dimensions under a common benchmark and local weather record, focusing detailed analysis on ventilation, screens, heating capacity, and sensor placement. In an operating greenhouse, it evaluates short-term temperature prediction and locates periods when passive measures or supplementary heating may be relevant. Translation into control commands requires a counterfactual model and controlled testing of the selected band.

The method requires fewer inputs than CFD, energy simulation, or model predictive control and can precede them when material properties, energy meters, and calibrated control models are limited (Lin et al., 2021; Chen et al., 2022; Mahmood et al., 2023; Narayanan et al., 2025; Nagarsheth et al., 2025; Labrozzi et al., 2026; Benzzine et al., 2025). A screened band could later serve as a constraint or test case for LSTM-based prediction or MPC (Benzzine et al., 2026, 2025). As a geometry-only descriptor, *C_G_* isolates structural susceptibility; detailed simulation or controlled trials incorporate glazing, leakage, radiation exchange, crop transpiration, and airflow.

*C_G_* is the optimal constant in the weighted Hardy-type inequality and, within an idealized diffusion comparison, relates to the slowest-decaying spatial mode. The fourth-root exponent follows from balancing *bC_G_*/*δ*^2^ against *gδ*^2^; it is not derived directly from greenhouse heat transfer or crop physiology. Controlled cross-geometry comparisons with spatial temperature fields and crop outcomes would estimate the tradeoff coefficients and test the proposed exponent.

### 4.3 Crop environment and production context

The high frequency of VPD below 0.5 kPa indicates weak atmospheric demand in much of the record, especially at night. Low or high VPD can affect transpiration, stomatal function, photosynthesis, and calcium transport, but the response depends on crop stage and management (Zhang et al., 2017; Cockshull, 1998). Air temperature and RH characterize atmospheric exposure, whereas attribution of physiological injury, condensation duration, disease, or fruit disorders requires measurements of leaf temperature, gas exchange, leaf wetness, and crop water use.

The fixed 25/18 °C temperature pair and 0.5–1.2 kPa VPD interval supported consistent late-stage comparison; future applications can use time-varying references based on phenology, radiation, daily light integral, and fruit load. The reported density of 1.25 plants/m^2^, productive stem density of 3.75 stems/m^2^, and annual yield of approximately 45 kg/m^2^ anchor the model in its commercial context, while treatment-specific yield response belongs to the prospective band comparison. Temperature-band screening is therefore one component of crop microclimate management and must be evaluated against the prevailing humidity and VPD environment.

### 4.4 Limitations and validation priorities

The study covers one Venlo compartment and a late-season period dominated by heating. Although two measuring boxes contributed to the compartment-level mean, only the averaged canopy-top series was available for analysis; the two individual series were not analyzed separately. The record therefore characterized the mean canopy-top air environment but could not resolve vertical or horizontal temperature gradients or validate the three-dimensional field assumed by the geometry formulation. More granular device and service metadata are retained in vendor-managed and site-specific technical records and were not available for public disclosure at the time of revision. The boundary model, weighting family, *α*, benchmark, and scaling exponents were declared rather than calibrated across greenhouses. Actuator records were generated endogenously by the existing controller, and no metered energy, cooling setpoint, treatment-specific crop response, or counterfactual temperature trajectory was available. These limitations restrict inference to preliminary scenario selection for this site and monitored period.

The next step is a controlled comparison of the current operating strategy and selected candidates, including the illustrative 1.21 °C midpoint. Temperature and humidity should be measured at several heights and locations together with heat, electricity, water flow, vent and screen states, canopy temperature, gas exchange, biomass allocation, yield, and fruit quality. Randomized or stepped band interventions are needed to identify counterfactual actuator and crop responses. Tests should cover heating- and cooling-dominated seasons and geometrically distinct greenhouses. These measurements would determine whether the descriptor and scaling hypothesis are transferable and whether a screened band reduces resource use without compromising the crop.

## 5 Conclusions

This study established a low input framework combining a spectral geometry descriptor, one hour temperature prediction, crop-environment exposure, and operating records to prioritize temperature-band hypotheses for field testing. Under the declared benchmark and preliminary fourth-root hypothesis, the Okawa calculation generated candidate half widths of 0.97–1.45 °C, with 1.21 °C as the conditional midpoint. The ARX holdout supplied complementary evidence for local temperature predictability; prospective cross-geometry trials will test the geometry-to-band relationship.

On the same observed trajectory, the 1.21 °C scenario lowered the retrospective degree-hour index by 9.17% and increased temperature-band membership from 21.02% to 25.22% relative to the 1.0 °C half width. This index difference defines a hypothesis for metered-energy testing rather than a measured saving. Low VPD remained common, showing that temperature flexibility must be evaluated with humidity and crop response. Actuator sensitivity also documented predictor redundancy under the existing control regime.

The practical output is a short list of candidate bands and a structured evaluation protocol. Controlled trials combining spatial climate, resource use, crop physiology, yield, and fruit quality can determine whether the scaling hypothesis is suitable for new or operating greenhouses.

## Data Availability

The raw data supporting the conclusions of this article will be made available by the authors, without undue reservation.
