# Supplementary material for: Geometry-informed temperature band screening for crop microclimate management in a commercial tomato greenhouse
Source: Front Plant Sci. 2026 Sep 29;17:1969599. doi: 10.3389/fpls.2026.1969599 (PMC13660015; doi:10.3389/fpls.2026.1969599)
Supplement: Supplementary file 1 [file DataSheet1.pdf]

Supplementary Material

The main article contains the definitions, equations, and results needed to understand the study. This file provides the detailed spectral formulation, stationary point derivation, numerical sensitivity tables, joint temperature–VPD distribution, bootstrap sensitivity, ARX coefficients and residual diagnostics, actuator diagnostics, and application sequence used to support reproducibility.

S1. Weighted Hardy-type inequality and spectral characterization

Let $\xi = z/H$ and $w(\xi) = \xi^{-2\alpha}$, where $0 < \alpha < 1/2$. This restriction ensures that w is integrable on $(0, 1)$ in the present formulation. The parameter α controls the relative emphasis placed on lower heights. Because no vertical temperature profiles were available, neither this power-law form nor α was calibrated from measurements. The quotient

$$\mathcal{R}[e] = \frac{\int_\Omega |\nabla e|^2 \, d\mathbf{x}}{\int_\Omega w(z/H) e^2 \, d\mathbf{x}} \tag{1}$$

has units of 1/m^2. Its minimum λ_1 defines $C_G = 1/\lambda_1$ in m^2. The weak eigenproblem is

$$\int_\Omega \nabla e \cdot \nabla \eta \, d\mathbf{x} = \lambda \int_\Omega w(z/H) e \eta \, d\mathbf{x}, \qquad e, \eta \in H_0^1(\Omega). \tag{2}$$

The Dirichlet space defines a common mathematical comparison and is not a physical greenhouse boundary condition model. The associated weighted Hardy-type inequality is

$$\int_\Omega w e^2 \, d\mathbf{x} \le C_G \int_\Omega |\nabla e|^2 \, d\mathbf{x} \tag{3}$$

Here, C_G is the optimal Hardy-type constant for the stated domain, weight, and function space. The Rayleigh quotient and generalized principal eigenvalue provide its spectral characterization. Within this comparison problem, C_G is the maximum weighted error amplitude per unit gradient energy. For an ideal diffusion operator, an inverse first eigenvalue is proportional to a slow relaxation scale after division by diffusivity. The formulation remains entirely within this weighted Hardy-type framework and does not include greenhouse heat-transfer coefficients, airflow, radiation, crop transpiration, or actuators.

S2. Rayleigh–Ritz matrices

Using the lowest horizontal modes and N vertical sine modes,

$$e_N = \sin\left(\frac{\pi x}{L_x}\right) \sin\left(\frac{\pi y}{L_y}\right) \sum_{n=1}^{N} c_n \sin\left(\frac{n\pi z}{H}\right). \tag{4}$$

Let $\mu_h = (\pi/L_x)^2 + (\pi/L_y)^2$. The generalized eigenproblem is $\mathbf{Kc} = \lambda \mathbf{Mc}$, where

$$K_{mn} = \frac{1}{2} \left[\left(\frac{n\pi}{H} \right)^2 + \mu_h \right] \delta_{mn}, \tag{5}$$

$$M_{mn} = \int_0^1 \xi^{-2\alpha} \sin(m\pi\xi) \sin(n\pi\xi) \, d\xi. \tag{6}$$

The implementation used 800 point Gauss–Legendre quadrature and a Cholesky transformation to a symmetric standard eigenproblem.

Because the weight depends only on relative height, the rectangular problem separates. The first horizontal sine mode minimizes the horizontal Laplacian contribution, and any higher horizontal index

strictly increases it. The horizontal factor is therefore exact within the stated Dirichlet formulation; only the vertical expansion is truncated numerically.

S3. Single mode expression

For $N = 1$, define $I_\alpha = \int_0^1 \xi^{-2\alpha} \sin^2(\pi\xi)\, d\xi$. Then

$$C_{G,1m} = \frac{2I_\alpha}{(\pi/L_x)^2 + (\pi/L_y)^2 + (\pi/H)^2}. \tag{7}$$

At $\alpha = 0$, $I_0 = 1/2$ and the standard first Dirichlet eigenvalue of a rectangular domain is recovered.

S4. Stationary point derivation of the fourth root scenario rule

The declared phenomenological objective is

$$J(\delta) = bC_G\delta^{-2} + g\delta^2, \qquad b > 0, \quad g > 0. \tag{8}$$

For $\delta > 0$, its first and second derivatives are

$$\frac{dJ}{d\delta} = -2bC_G\delta^{-3} + 2g\delta, \tag{9}$$

$$\frac{d^2J}{d\delta^2} = 6bC_G\delta^{-4} + 2g > 0. \tag{10}$$

The unique stationary point is therefore a minimum and satisfies

$$-2bC_G\delta^{-3} + 2g\delta = 0 \quad \Longrightarrow \quad \delta^4 = \frac{bC_G}{g} \quad \Longrightarrow \quad \delta^* = \left(\frac{bC_G}{g}\right)^{1/4}. \tag{11}$$

If the declared coefficient ratio b/g is held constant for a site and benchmark pair, division gives

$$\frac{\delta_{\text{site}}}{\delta_{\text{b}}} = \left(\frac{C_{G,\text{site}}}{C_{G,\text{b}}}\right)^{1/4}. \tag{12}$$

The inverse square and quadratic terms encode opposing monotone screening preferences. The term C_G/δ^2 scales the spectral susceptibility by the squared permitted temperature deviation, whereas δ^2 penalizes greater tolerance. They are not measurements of a heat requirement, spatial temperature field, or crop physiological response, and the stationary point is not an empirically calibrated agronomic optimum.

The exponent is conditional on the selected powers. For the general screening objective

$$J_{p,q}(\delta) = bC_G\delta^{-p} + g\delta^q, \qquad p, q > 0, \tag{13}$$

the stationary condition gives

$$\delta^{p+q} = \frac{pbC_G}{qg}, \tag{14}$$

so the geometry exponent is $1/(p+q)$. The fourth root follows only from $p = q = 2$. It is therefore a preliminary scenario-generation hypothesis rather than a heat-transfer law, crop-response relationship, or validated control rule.

S5. Dependence on the declared benchmark geometry

To isolate normalization dependence, the reference half width was held at 1.0 °C while square benchmark plan area and height were varied. These cases are diagnostics rather than proposed standards. The resulting 1.047–1.290 range is scenario dependence on the reporting pair, not prediction error or statistical bias. Height had the larger influence over this diagnostic grid. Cross study comparisons should report benchmark dimensions and the associated reference half width together.

Table S1: Sensitivity of the fourth root multiplier to the declared benchmark geometry at $\alpha = 0.30$. The row used in the main analysis is shown in bold.

Area, m^2	Height, m	$C_{G,\mathrm{b}}$, m^2	Multiplier	Site half width, °C
500	4.0	2.596	1.290	1.290
500	4.5	3.235	1.221	1.221
500	6.0	5.445	1.072	1.072
1000	4.0	2.674	1.281	1.281
**1000**	**4.5**	**3.357**	**1.210**	**1.210**
1000	6.0	5.798	1.055	1.055
2000	4.0	2.715	1.276	1.276
2000	4.5	3.422	1.204	1.204
2000	6.0	5.993	1.047	1.047

S6. Mode convergence for the Okawa geometry

Table S2: Vertical mode convergence of C_G at $\alpha = 0.30$.

Vertical modes N	C_G, m^2
4	7.185156
8	7.188040
12	7.188258
24	7.188318
48	7.188323

The 24-mode value differs from the 48-mode value by less than 0.001%. The submitted reproducibility code also evaluates single mode error across the geometry and α grid reported in the manuscript.

S7. Crop environment exposure calculation

Each five minute observation was assigned a day and night reference temperature of 25/18 °C. This literature-guided pair was an exposure reference rather than a reconstruction of the commercial climate-computer settings or a crop-stage-specific optimum. For half width δ, temperature-band membership required $T_{\mathrm{ref}} - \delta \leq T_g \leq T_{\mathrm{ref}} + \delta$. VPD was calculated from greenhouse air temperature and RH. The literature-guided 0.5–1.2 kPa interval was used only as an atmospheric screening range. Joint membership required both temperature-band and VPD membership. Exposure was summarized for daytime (06:00–18:00), night (18:00–06:00), and all 24 h. The VPD screening interval contained 28.38% of daytime records, 0.15% of night records, and 14.28% of all records. Joint membership for the declared-reference and illustrative bands was 12.55% and 14.61% during daytime, and approximately 0.02% for both bands at night. No membership statistic was interpreted as a physiological, yield, or quality response.

S8. Joint temperature and VPD distribution

S9. Calendar-day and moving-block bootstrap sensitivity

Five minute degree-hour contributions were aggregated by calendar day before resampling. This preserves dependence among the 288 within-day observations but treats resampled days as exchangeable. The lag-1 autocorrelation of the paired daily index difference was 0.526, indicating that dependence between consecutive days was not negligible. A seven-day moving-block bootstrap was therefore added. Only blocks containing seven consecutive complete calendar days were eligible; 71 starting positions were

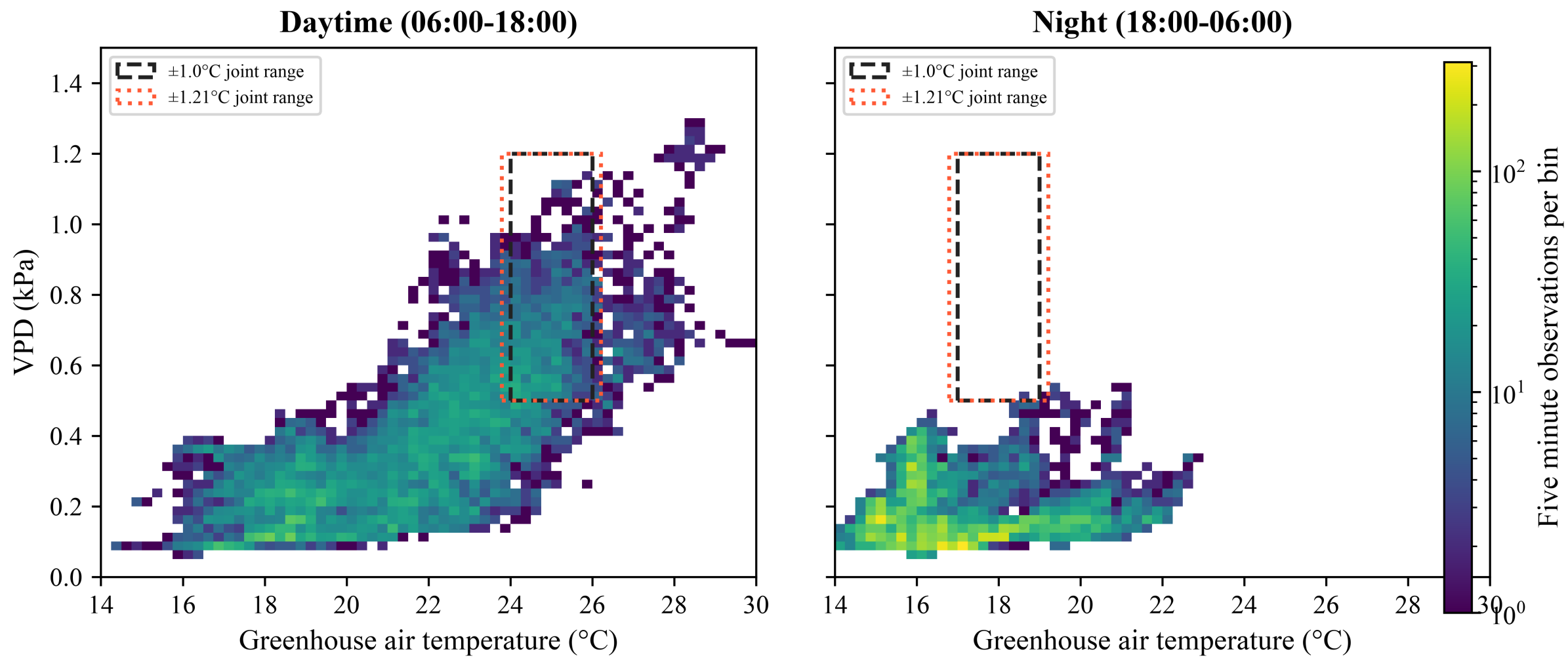

Figure S1: Daytime and night joint distributions of greenhouse air temperature and VPD. Black dashed rectangles show membership in the declared-reference temperature band and the 0.5–1.2 kPa VPD interval; orange dotted rectangles show the illustrative wider temperature band with the same VPD interval. Bin color uses a logarithmic count scale. The rectangles are declared screening ranges, not physiological optima.

available. Each procedure used 20,000 resamples and a fixed seed. The calendar-day 95% percentile interval for the relative index change was 8.66–9.68%; the seven-day moving-block interval was 8.41–10.14%. The latter is reported as a robustness check rather than evidence of energy saving.

S10. ARX coefficients and residual diagnostics

The ARX model was fitted by ordinary least squares on W37–W44. Coefficients use the recorded scales; radiation is the hourly mean of the weather-station channel. Because predictors were not standardized, coefficient magnitudes should not be compared as measures of importance.

Table S3: Fitted ARX coefficients for the W37–W44 calibration period.

Term	Coefficient
Intercept	2.406767
Previous greenhouse temperature	0.858295
Outdoor temperature	0.018368
Solar radiation	0.000754
$\sin(2\pi q/24)$	0.928082
$\cos(2\pi q/24)$	−0.273951

Residuals were defined as predicted minus observed temperature and retained in chronological order. The nonzero 1 h and 24 h autocorrelations show that the simple model did not remove all short-term and diurnal structure.

Table S4: Descriptive diagnostics for ordered one-step ARX residuals. Temperature summaries are in °C.

Period	Mean	SD	Median	P05	P95	Durbin–Watson	ACF 1 h	ACF 6 h	ACF 12 h	ACF 24 h
Calibration, W37–W44	0.000	0.788	0.018	−1.225	1.136	1.096	0.452	−0.227	0.265	0.525
Temporal holdout, W45–W48	0.029	0.798	0.080	−1.429	1.256	1.392	0.302	−0.237	0.096	0.486

These diagnostics qualify the predictive result; they do not invalidate the reported RMSE comparison, but they show that the ARX specification is not a complete dynamic model. The ARX contains no C_G term and therefore provides no validation of the geometry-to-band scaling.

S11. ARX sensitivity with actuator inputs

During W38–W44, hourly rows with complete vent, screen, heating water, and environmental channels were retained. Base, passive-channel, and passive-and-heating ARX specifications were calibrated on W38–W42 and evaluated on W43–W44. The passive model added mean north and south vent opening and two screen deployment channels; the full model also added heating water temperature. Metrics quantify within-site temporal prediction only. Because the recorded actuator states were generated by the existing controller and management in response to the same climate, their coefficients and performance differences are subject to endogeneity and are not causal estimates of actuator effects.

Table S5: Multicollinearity diagnostics for the W38–W42 calibration predictors.

Specification	Condition number	Maximum $\lvert r \rvert$	Pair	Maximum VIF
Base ARX	6.92	0.780	Indoor temperature, cosine term	10.92
Passive-channel ARX	9.05	0.933	Screen 1, screen 2	11.51
Passive-and-heating ARX	9.12	0.933	Screen 1, screen 2	11.51

Screen 1 and screen 2 had VIF values of 9.25–9.91. The diagnostics identify substantial predictor redundancy that can destabilize incremental coefficients, but they neither correct the endogeneity of controller-generated actuator states nor establish why a particular holdout difference occurred.

S12. Evidence levels and application procedure

The application sequence is: (1) enter greenhouse length, width, height, and a declared reference band family; (2) calculate C_G and verify numerical convergence; (3) apply the preliminary scaling hypothesis to generate a candidate band family; (4) calibrate the ARX model with local records and evaluate it on a chronological holdout; (5) calculate degree-hour indices and temperature, RH, and VPD exposure on a common observed trajectory; (6) assess whether vent, screen, and supplementary heating records provide physically plausible operating pathways; and (7) transfer the retained candidate family to prospective tests with spatial climate, direct energy, water, physiology, yield, and quality measurements.

Numerical convergence verifies only the calculation of C_G within the specified mathematical formulation. The temporal holdout evaluates only the ARX for the observed compartment and crop cycle. The operating checks establish associations, not causal actuator effects. None of these steps validates the fourth-root scaling hypothesis. Validation of that relationship requires prospective comparison of candidate bands across greenhouse geometries and seasons.
